# *N*-Methyl-4-(4-pivalamido­phenyl­sulfan­yl)picolinamide hemihydrate

**DOI:** 10.1107/S1600536811012268

**Published:** 2011-04-13

**Authors:** Ting-Hong Ye, Ting-Ting Huang, Yao-Jie Shi, Ying Xiong, Luo-Ting Yu

**Affiliations:** a State Key Laboratory of Biotherapy and Cancer Center, West China Hospital, West China Medical School, Sichuan University, Chengdu 610041, People’s Republic of China; b West China School of Pharmacy, Sichuan University, Chengdu 610041, People’s Republic of China

## Abstract

In the title compound, C_18_H_21_N_3_O_2_S·0.5H_2_O, the benzene ring makes dihedral angles of 88.59 (6) and 40.74 (8)° with the pyridine ring and the amide group, respectively. The water O atom lies on a twofold axis. In the crystal, the organic mol­ecules and the water mol­ecules are linked *via* O—H⋯O hydrogen bonds, while the organic mol­ecules are connected to each other *via* N—H⋯O hydrogen bonds, forming a three-dimensional network.

## Related literature

For related compounds and their biological activity, see: Khire *et al.* (2004 ▶); Dominguez *et al.* (2007 ▶).
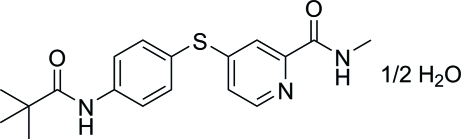


## Experimental

### 

#### Crystal data

C_18_H_21_N_3_O_2_S·0.5H_2_O
*M* *_r_* = 352.46
Monoclinic, 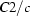

*a* = 12.7413 (4) Å
*b* = 17.5056 (8) Å
*c* = 17.1978 (6) Å
β = 108.632 (4)°
*V* = 3634.8 (2) Å^3^
*Z* = 8
Mo *K*α radiation
μ = 0.20 mm^−1^
*T* = 293 K
0.24 × 0.22 × 0.18 mm

#### Data collection

Oxford Diffraction Xcalibur Eos diffractometer
Absorption correction: multi-scan (*CrysAlis PRO*; Oxford Diffraction, 2010 ▶) *T*_min_ = 0.966, *T*_max_ = 1.000
3714 measured reflections
3714 independent reflections
2498 reflections with *I* > 2σ(*I*)

#### Refinement

*R*[*F* ^2^ > 2σ(*F* ^2^)] = 0.040
*wR*(*F* ^2^) = 0.111
*S* = 0.96
3714 reflections
238 parameters
H atoms treated by a mixture of independent and constrained refinement
Δρ_max_ = 0.25 e Å^−3^
Δρ_min_ = −0.21 e Å^−3^

### 

Data collection: *CrysAlis PRO* (Oxford Diffraction, 2010 ▶); cell refinement: *CrysAlis PRO*; data reduction: *CrysAlis PRO*; program(s) used to solve structure: *SHELXS97* (Sheldrick, 2008 ▶); program(s) used to refine structure: *SHELXL97* (Sheldrick, 2008 ▶); molecular graphics: *OLEX2* (Dolomanov *et al.*, 2009 ▶) and Mercury (Macrae *et al.*, 2006 ▶); software used to prepare material for publication: *OLEX2*.

## Supplementary Material

Crystal structure: contains datablocks I, global. DOI: 10.1107/S1600536811012268/zq2092sup1.cif

Structure factors: contains datablocks I. DOI: 10.1107/S1600536811012268/zq2092Isup2.hkl

Additional supplementary materials:  crystallographic information; 3D view; checkCIF report

## Figures and Tables

**Table 1:** Hydrogen-bond geometry (Å, °)

*D*—H⋯*A*	*D*—H	H⋯*A*	*D*⋯*A*	*D*—H⋯*A*
N3—H3*A*⋯O1^i^	0.854 (18)	2.335 (18)	3.0317 (19)	139.0 (15)
O3—H3⋯O2^ii^	0.87 (2)	1.94 (2)	2.8059 (18)	174.2
N1—H1⋯O3	0.850 (19)	2.269 (19)	3.089 (2)	162.3 (18)

Symmetry codes: (i) 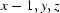; (ii) 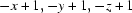.

**Fig. 1.**
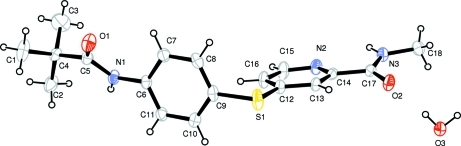
The molecular structure of the title compound, with displacement ellipsoids drawn at the 50% probability level.

**Fig. 2.**
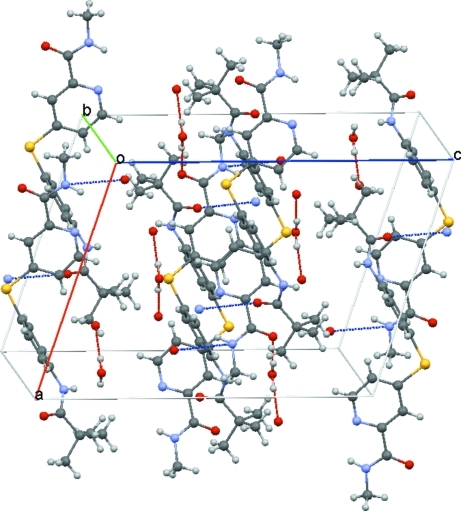
A packing diagram of the title compound, with O–H···O and N–H···O hydrogen bonds shown as dotted red and blue lines, respectively.

## supplementary crystallographic information

### Comment

Sorafenib is a molecule of great importance owing to its antitumor properties (Khire *et al.*, 2004; Dominguez *et al.*, 2007). The title compound, which is one of its derivatives, possesses even better *in vitro* anticancer activity against both two tumor cell lines (HCT116 and HEPG2). As a potent antitumor drug, we report here its crystal structure.

In the title molecule, C_18_H_21_N_3_O_2_S.0.5(H_2_O), the central benzene ring makes dihedral angles of 88.59 (6)° and 40.74 (8)° with the pyridine and amide bonds, respectively (Fig. 1). The O atom of the isolated water molecule lies on a two-fold axis. In the crystal structure, two organic molecules and one water molecule are linked *via* the O3—H3···O2^ii^ hydrogen bonds, while organic molecules are connected with each other *via* the N3—H3A···O1^i^ hydrogen bonds, forming a three-dimensional network [symmetry codes: (i) *x*-1, *y*, *z*; (ii) *x*+3/2, *y*+3/2, *z*+1.Table 1 and Fig. 2].

### Experimental

To the suspension of anhydrous potassium carbonate (0.69 g, 5.0 mmol) and 4-(4-aminophenylthio)-*N*-methylpicolinamide (0.52 g, 2.0 mmol) in 7.0 ml THF was added dropwise pivaloyl chloride (0.25 g, 2.1 mmol). After being stirred at room temperature for 2 h, the mixture was extracted with 30 ml EA and 30 ml brine for three times and the combined organic layers were dried over anhydrous sodium sulfate. Then the solution was concentrated under vacuum, and the residue was recrystallized from ethanol to give the title compound, with 40% yield. Crystals suitable for a X-ray analysis were obtained by slow evaporation from a solution of ethanol at room temperature.

### Refinement

The two H atoms of N1 and N3 were located in a difference Fourier map and refined isotropically. The remaining H atoms were positioned geometrically (C—H = 0.93–0.96 Å) and refined using a riding model, with *U*_iso_(H) = 1.2–1.5*U*_eq_(C).

### Crystal data

**Table:**

C_18_H_21_N_3_O_2_S·0.5H_2_O	*F*(000) = 1496
*M**_r_* = 352.46	*D*_x_ = 1.288 Mg m^−^^3^
Monoclinic, *C*2/*c*	Mo *K*α radiation, λ = 0.7107 Å
Hall symbol: -C 2yc	Cell parameters from 4009 reflections
*a* = 12.7413 (4) Å	θ = 3.2–28.9°
*b* = 17.5056 (8) Å	µ = 0.20 mm^−^^1^
*c* = 17.1978 (6) Å	*T* = 293 K
β = 108.632 (4)°	Block, colourless
*V* = 3634.8 (2) Å^3^	0.24 × 0.22 × 0.18 mm
*Z* = 8	

### Data collection

**Table:**

Oxford Diffraction Xcalibur Eos diffractometer	3714 independent reflections
Radiation source: fine-focus sealed tube	2498 reflections with *I* > 2σ(*I*)
graphite	*R*_int_ = 0.000
Detector resolution: 16.0874 pixels mm^-1^	θ_max_ = 26.4°, θ_min_ = 3.4°
ω scans	*h* = −15→15
Absorption correction: multi-scan (*CrysAlis PRO*; Oxford Diffraction, 2010)	*k* = 0→21
*T*_min_ = 0.966, *T*_max_ = 1.000	*l* = 0→21
3714 measured reflections	

### Refinement

**Table:**

Refinement on *F*^2^	Primary atom site location: structure-invariant direct methods
Least-squares matrix: full	Secondary atom site location: difference Fourier map
*R*[*F*^2^ > 2σ(*F*^2^)] = 0.040	Hydrogen site location: inferred from neighbouring sites
*wR*(*F*^2^) = 0.111	H atoms treated by a mixture of independent and constrained refinement
*S* = 0.96	*w* = 1/[σ^2^(*F*_o_^2^) + (0.0661*P*)^2^] where *P* = (*F*_o_^2^ + 2*F*_c_^2^)/3
3714 reflections	(Δ/σ)_max_ = 0.001
238 parameters	Δρ_max_ = 0.25 e Å^−^^3^
0 restraints	Δρ_min_ = −0.21 e Å^−^^3^

### Special details

**Table:**

Experimental. CrysAlisPro, Oxford Diffraction Ltd. (version 1.171.33.66). Empirical absorption correction using spherical harmonics, implemented in SCALE3 ABSPACK scaling algorithm.
Geometry. All e.s.d.'s (except the e.s.d. in the dihedral angle between two l.s. planes) are estimated using the full covariance matrix. The cell e.s.d.'s are taken into account individually in the estimation of e.s.d.'s in distances, angles and torsion angles; correlations between e.s.d.'s in cell parameters are only used when they are defined by crystal symmetry. An approximate (isotropic) treatment of cell e.s.d.'s is used for estimating e.s.d.'s involving l.s. planes.
Refinement. Refinement of *F*^2^ against ALL reflections. The weighted *R*-factor *wR* and goodness of fit *S* are based on *F*^2^, conventional *R*-factors *R* are based on *F*, with *F* set to zero for negative *F*^2^. The threshold expression of *F*^2^ > σ(*F*^2^) is used only for calculating *R*-factors(gt) *etc*. and is not relevant to the choice of reflections for refinement. *R*-factors based on *F*^2^ are statistically about twice as large as those based on *F*, and *R*- factors based on ALL data will be even larger.

### Fractional atomic coordinates and isotropic or equivalent isotropic displacement parameters (Å^2^)

**Table:**

	*x*	*y*	*z*	*U*_iso_*/*U*_eq_	
S1	0.61494 (4)	0.59213 (4)	0.36246 (3)	0.0608 (2)	
O1	1.13699 (9)	0.68403 (8)	0.59009 (7)	0.0587 (4)	
O2	0.18072 (9)	0.62917 (8)	0.30133 (7)	0.0501 (3)	
N1	1.04402 (11)	0.59473 (9)	0.63584 (9)	0.0426 (4)	
N2	0.36966 (11)	0.62678 (10)	0.50293 (8)	0.0467 (4)	
N3	0.15906 (11)	0.66751 (9)	0.42051 (9)	0.0437 (4)	
C1	1.33721 (15)	0.64847 (17)	0.70547 (13)	0.0802 (8)	
H1B	1.3331	0.6977	0.6800	0.120*	
H1A	1.3476	0.6098	0.6690	0.120*	
H1C	1.3984	0.6476	0.7556	0.120*	
C2	1.23823 (17)	0.55645 (13)	0.76612 (13)	0.0702 (7)	
H2C	1.3062	0.5537	0.8111	0.105*	
H2A	1.2368	0.5165	0.7276	0.105*	
H2B	1.1767	0.5505	0.7864	0.105*	
C3	1.2132 (2)	0.69544 (16)	0.78024 (13)	0.0901 (8)	
H3C	1.2060	0.7439	0.7528	0.135*	
H3D	1.2756	0.6969	0.8296	0.135*	
H3B	1.1471	0.6850	0.7938	0.135*	
C4	1.23043 (13)	0.63306 (10)	0.72414 (10)	0.0412 (4)	
C5	1.13433 (13)	0.63957 (11)	0.64372 (10)	0.0397 (4)	
C6	0.94575 (12)	0.59550 (10)	0.56813 (10)	0.0382 (4)	
C7	0.89869 (14)	0.66307 (11)	0.53038 (11)	0.0458 (4)	
H7	0.9337	0.7095	0.5478	0.055*	
C8	0.79980 (14)	0.66091 (12)	0.46693 (10)	0.0481 (5)	
H8	0.7689	0.7062	0.4415	0.058*	
C9	0.74604 (13)	0.59274 (12)	0.44062 (10)	0.0446 (5)	
C10	0.79331 (13)	0.52514 (12)	0.47770 (10)	0.0479 (5)	
H10	0.7582	0.4787	0.4601	0.058*	
C11	0.89264 (13)	0.52707 (11)	0.54086 (10)	0.0444 (4)	
H11	0.9243	0.4816	0.5654	0.053*	
C12	0.52341 (13)	0.60419 (10)	0.41985 (10)	0.0399 (4)	
C13	0.41182 (12)	0.61603 (10)	0.37742 (9)	0.0381 (4)	
H13	0.3863	0.6161	0.3204	0.046*	
C14	0.34005 (12)	0.62763 (10)	0.42101 (9)	0.0366 (4)	
C15	0.47617 (14)	0.61313 (12)	0.54174 (11)	0.0559 (5)	
H15	0.4987	0.6107	0.5987	0.067*	
C16	0.55535 (14)	0.60244 (12)	0.50456 (10)	0.0507 (5)	
H16	0.6289	0.5942	0.5356	0.061*	
C17	0.21897 (12)	0.64171 (10)	0.37579 (10)	0.0382 (4)	
C18	0.04265 (13)	0.68624 (11)	0.38587 (11)	0.0509 (5)	
H18B	0.0278	0.7041	0.3306	0.076*	
H18C	0.0240	0.7254	0.4183	0.076*	
H18A	−0.0010	0.6415	0.3858	0.076*	
H3A	0.1891 (14)	0.6747 (11)	0.4720 (11)	0.051 (5)*	
H1	1.0476 (15)	0.5594 (11)	0.6705 (11)	0.056 (6)*	
H3	0.9464 (16)	0.4381 (12)	0.7371 (14)	0.072 (7)*	
O3	1.0000	0.47126 (12)	0.7500	0.0502 (5)	

### Atomic displacement parameters (Å^2^)

**Table:**

	*U*^11^	*U*^22^	*U*^33^	*U*^12^	*U*^13^	*U*^23^
S1	0.0340 (2)	0.1137 (5)	0.0331 (2)	0.0111 (3)	0.00870 (18)	0.0027 (3)
O1	0.0487 (7)	0.0767 (10)	0.0474 (7)	−0.0134 (7)	0.0109 (6)	0.0197 (7)
O2	0.0346 (6)	0.0727 (9)	0.0386 (7)	−0.0011 (6)	0.0059 (5)	−0.0086 (6)
N1	0.0340 (7)	0.0498 (10)	0.0401 (8)	−0.0034 (7)	0.0061 (6)	0.0113 (8)
N2	0.0369 (7)	0.0677 (11)	0.0349 (8)	−0.0007 (7)	0.0106 (6)	−0.0011 (7)
N3	0.0350 (7)	0.0564 (10)	0.0389 (8)	0.0019 (7)	0.0107 (7)	−0.0019 (7)
C1	0.0398 (10)	0.134 (2)	0.0608 (13)	−0.0199 (12)	0.0076 (10)	0.0188 (14)
C2	0.0557 (11)	0.0722 (16)	0.0628 (13)	−0.0126 (11)	−0.0090 (10)	0.0177 (12)
C3	0.105 (2)	0.100 (2)	0.0551 (14)	0.0152 (16)	0.0122 (14)	−0.0235 (13)
C4	0.0380 (9)	0.0493 (11)	0.0338 (9)	−0.0092 (8)	0.0082 (7)	−0.0009 (8)
C5	0.0370 (9)	0.0459 (11)	0.0375 (9)	−0.0023 (8)	0.0139 (7)	0.0013 (8)
C6	0.0308 (8)	0.0478 (11)	0.0370 (9)	0.0005 (8)	0.0121 (7)	0.0052 (8)
C7	0.0409 (9)	0.0453 (11)	0.0507 (10)	0.0015 (8)	0.0138 (8)	0.0057 (9)
C8	0.0416 (9)	0.0563 (12)	0.0475 (10)	0.0138 (9)	0.0159 (8)	0.0157 (9)
C9	0.0300 (8)	0.0690 (13)	0.0352 (9)	0.0052 (9)	0.0108 (7)	0.0052 (9)
C10	0.0386 (9)	0.0567 (13)	0.0458 (10)	−0.0040 (9)	0.0098 (8)	−0.0017 (9)
C11	0.0368 (9)	0.0451 (11)	0.0471 (10)	0.0023 (8)	0.0073 (8)	0.0090 (8)
C12	0.0323 (8)	0.0511 (11)	0.0347 (9)	0.0008 (7)	0.0083 (7)	−0.0002 (8)
C13	0.0326 (8)	0.0485 (11)	0.0295 (8)	−0.0005 (7)	0.0048 (7)	−0.0007 (7)
C14	0.0323 (8)	0.0392 (10)	0.0351 (9)	−0.0035 (7)	0.0065 (7)	−0.0043 (8)
C15	0.0404 (9)	0.0945 (17)	0.0300 (9)	0.0005 (10)	0.0071 (7)	0.0001 (9)
C16	0.0340 (8)	0.0802 (15)	0.0331 (9)	0.0042 (9)	0.0042 (7)	0.0015 (9)
C17	0.0315 (8)	0.0397 (10)	0.0422 (10)	−0.0042 (7)	0.0102 (7)	−0.0014 (8)
C18	0.0380 (9)	0.0586 (13)	0.0585 (12)	0.0060 (9)	0.0189 (8)	0.0009 (10)
O3	0.0371 (10)	0.0505 (12)	0.0565 (11)	0.000	0.0056 (9)	0.000

### Geometric parameters (Å, °)

**Table:**

S1—C9	1.7783 (16)	C4—C5	1.531 (2)
S1—C12	1.7646 (17)	C6—C7	1.390 (2)
O1—C5	1.215 (2)	C6—C11	1.382 (2)
O2—C17	1.2355 (18)	C7—H7	0.9300
N1—C5	1.364 (2)	C7—C8	1.379 (2)
N1—C6	1.412 (2)	C8—H8	0.9300
N1—H1	0.850 (19)	C8—C9	1.378 (3)
N2—C14	1.337 (2)	C9—C10	1.387 (3)
N2—C15	1.329 (2)	C10—H10	0.9300
N3—C17	1.324 (2)	C10—C11	1.380 (2)
N3—C18	1.448 (2)	C11—H11	0.9300
N3—H3A	0.854 (18)	C12—C13	1.391 (2)
C1—H1B	0.9600	C12—C16	1.382 (2)
C1—H1A	0.9600	C13—H13	0.9300
C1—H1C	0.9600	C13—C14	1.370 (2)
C1—C4	1.519 (2)	C14—C17	1.510 (2)
C2—H2C	0.9600	C15—H15	0.9300
C2—H2A	0.9600	C15—C16	1.369 (2)
C2—H2B	0.9600	C16—H16	0.9300
C2—C4	1.511 (3)	C18—H18B	0.9600
C3—H3C	0.9600	C18—H18C	0.9600
C3—H3D	0.9600	C18—H18A	0.9600
C3—H3B	0.9600	O3—H3	0.87 (2)
C3—C4	1.519 (3)		
			
O1—C5—N1	121.37 (15)	C6—N1—H1	114.8 (13)
O1—C5—C4	121.67 (15)	C6—C7—H7	120.2
O2—C17—N3	123.57 (14)	C6—C11—H11	119.5
O2—C17—C14	120.31 (14)	C7—C6—N1	122.01 (16)
N1—C5—C4	116.93 (14)	C7—C8—H8	119.5
N2—C14—C13	124.18 (14)	C8—C7—C6	119.68 (17)
N2—C14—C17	116.27 (14)	C8—C7—H7	120.2
N2—C15—H15	117.4	C8—C9—S1	120.11 (14)
N2—C15—C16	125.22 (16)	C8—C9—C10	119.37 (15)
N3—C17—C14	116.12 (14)	C9—C8—C7	121.07 (16)
N3—C18—H18B	109.5	C9—C8—H8	119.5
N3—C18—H18C	109.5	C9—C10—H10	120.2
N3—C18—H18A	109.5	C10—C9—S1	120.48 (15)
C1—C4—C3	109.12 (19)	C10—C11—C6	120.98 (16)
C1—C4—C5	107.93 (13)	C10—C11—H11	119.5
H1B—C1—H1A	109.5	C11—C6—N1	118.74 (15)
H1B—C1—H1C	109.5	C11—C6—C7	119.20 (15)
H1A—C1—H1C	109.5	C11—C10—C9	119.68 (17)
C2—C4—C1	109.39 (17)	C11—C10—H10	120.2
C2—C4—C3	109.59 (18)	C12—S1—C9	101.86 (7)
C2—C4—C5	114.16 (14)	C12—C13—H13	120.5
H2C—C2—H2A	109.5	C12—C16—H16	120.8
H2C—C2—H2B	109.5	C13—C12—S1	118.16 (12)
H2A—C2—H2B	109.5	C13—C14—C17	119.55 (14)
C3—C4—C5	106.51 (15)	C14—C13—C12	118.96 (14)
H3C—C3—H3D	109.5	C14—C13—H13	120.5
H3C—C3—H3B	109.5	C15—N2—C14	115.50 (14)
H3D—C3—H3B	109.5	C15—C16—C12	118.47 (15)
C4—C1—H1B	109.5	C15—C16—H16	120.8
C4—C1—H1A	109.5	C16—C12—S1	124.21 (12)
C4—C1—H1C	109.5	C16—C12—C13	117.62 (15)
C4—C2—H2C	109.5	C16—C15—H15	117.4
C4—C2—H2A	109.5	C17—N3—C18	122.88 (15)
C4—C2—H2B	109.5	C17—N3—H3A	120.1 (12)
C4—C3—H3C	109.5	C18—N3—H3A	117.0 (12)
C4—C3—H3D	109.5	H18B—C18—H18C	109.5
C4—C3—H3B	109.5	H18B—C18—H18A	109.5
C5—N1—C6	125.06 (15)	H18C—C18—H18A	109.5
C5—N1—H1	119.7 (13)		
			
S1—C9—C10—C11	−176.99 (13)	C7—C8—C9—S1	176.56 (13)
S1—C12—C13—C14	−178.20 (13)	C7—C8—C9—C10	−1.1 (3)
S1—C12—C16—C15	179.54 (16)	C8—C9—C10—C11	0.7 (3)
N1—C6—C7—C8	−176.92 (15)	C9—S1—C12—C13	172.12 (14)
N1—C6—C11—C10	176.57 (15)	C9—S1—C12—C16	−8.07 (19)
N2—C14—C17—O2	−166.19 (16)	C9—C10—C11—C6	0.3 (3)
N2—C14—C17—N3	13.7 (2)	C11—C6—C7—C8	0.4 (3)
N2—C15—C16—C12	−1.4 (3)	C12—S1—C9—C8	−87.13 (15)
C1—C4—C5—O1	−32.9 (2)	C12—S1—C9—C10	90.55 (15)
C1—C4—C5—N1	148.93 (18)	C12—C13—C14—N2	−1.5 (3)
C2—C4—C5—O1	−154.74 (18)	C12—C13—C14—C17	179.11 (15)
C2—C4—C5—N1	27.1 (2)	C13—C12—C16—C15	−0.6 (3)
C3—C4—C5—O1	84.2 (2)	C13—C14—C17—O2	13.2 (2)
C3—C4—C5—N1	−94.0 (2)	C13—C14—C17—N3	−166.83 (16)
C5—N1—C6—C7	−41.0 (2)	C14—N2—C15—C16	1.9 (3)
C5—N1—C6—C11	141.63 (17)	C15—N2—C14—C13	−0.4 (3)
C6—N1—C5—O1	−1.4 (3)	C15—N2—C14—C17	178.99 (17)
C6—N1—C5—C4	176.82 (15)	C16—C12—C13—C14	2.0 (2)
C6—C7—C8—C9	0.6 (3)	C18—N3—C17—O2	−2.2 (3)
C7—C6—C11—C10	−0.9 (3)	C18—N3—C17—C14	177.90 (15)

### Hydrogen-bond geometry (Å, °)

**Table:**

*D*—H···*A*	*D*—H	H···*A*	*D*···*A*	*D*—H···*A*
N3—H3A···O1^i^	0.854 (18)	2.335 (18)	3.0317 (19)	139.0 (15)
O3—H3···O2^ii^	0.87 (2)	1.94 (2)	2.8059 (18)	174.2
N1—H1···O3	0.850 (19)	2.269 (19)	3.089 (2)	162.3 (18)

Symmetry codes: (i) *x*−1, *y*, *z*; (ii) −*x*+1, −*y*+1, −*z*+1.
